# Qizhi Kebitong Formula Ameliorates Sciatic Nerve Injury in Streptozocin-induced Diabetic Mice through PERK/ATF4/CHOP Endoplasmic Reticulum Stress Signaling Pathway

**DOI:** 10.2174/0113816128362557250314054528

**Published:** 2025-03-21

**Authors:** Honghai Yu, Cunqing Yang, Guoqiang Wang, Jiao Lv, Xiangyan Li, Wenxiu Qi, Xiuge Wang

**Affiliations:** 1 College of Traditional Chinese Medicine, Changchun University of Chinese Medicine, Changchun, Jilin, 130117, China;; 2 Department of Endocrinology, Guang’anmen Hospital, China Academy of Chinese Medical Sciences, Beijing, 100053, China;; 3 Department of Endocrinology, Affiliated Hospital of Changchun University of Chinese Medicine, Changchun, Jilin, 130012, China;; 4 Key Laboratory of Active Substances and Biological Mechanisms of Ginseng Efficacy, Key Laboratory of Bio-Macromolecules of Chinese Medicine, Ministry of Education, Jilin Provincial, Northeast Asia Research Institute of Traditional Chinese Medicine, Changchun University of Chinese Medicine, Changchun, Jilin, 130117, China

**Keywords:** Diabetic peripheral neuropathy, Qizhi Kebitong formula, UHPLC/QTOF-MS, endoplasmic reticulum stress, PERK/ATF4/CHOP pathway, diabetes mellitus

## Abstract

**Background:**

The Qizhi Kebitong formula (QKF) has been utilized as a traditional Chinese medicine (TCM) remedy for over two decades in treating diabetic peripheral neuropathy (DPN) with notable clinical efficacy. However, its precise mechanism and bioactive constituents remain elusive.

**Methods:**

Through ultra-high performance liquid chromatography-quadrupole time-of-flight mass spectrometry (UHPLC/QTOF-MS) analysis was used to identify the primary components of QKF. Nerve conduction function in mice was assessed by measuring sensory thresholds and nerve conduction velocities. Laser speckle contrast imaging (LSCI) was used to examine the effect of QKF on foot pads and perineural blood flow in mice. Additionally, Transmission electron microscopy (TEM) and various pathologic stains were utilized to observe QKF's therapeutic effect on sciatic nerve (SN) damage in DPN mice. The impact of QKF on the pathological mechanism of the DPN model was explored through qRT-PCR, Western blot, and immunohistochemistry.

**Results:**

Our results demonstrated that QKF improved phenotypic features in a mouse model of DPN, increased blood flow around the foot pad and SN, and somewhat repaired the pathological structure and function of SN. Furthermore, the study revealed that QKF slowed down the progression of DPN by inhibiting the endoplasmic reticulum (ER) stress apoptosis signaling pathway mediated by PERK/ATF4/CHOP pathway.

**Conclusion:**

The significant neuroprotective effects of QKF in experimental DPN mice were confirmed by our findings, which offer important scientific evidence supporting its potential utilization in DPN treatment.

## 1 INTRODUCTION

Diabetes mellitus (DM) is a global health concern, impacting 422 million individuals worldwide, and it is projected that by 2045, the number of people with DM will increase to 693 million [1, 2]. Diabetic peripheral neuropathy (DPN) is a common chronic complication of DM, affecting more than half of individuals with the condition. It is characterized by progressive nerve damage that starts from the distal regions and extends towards the proximal areas [3, 4]. In the early stages, sensory fiber damage in the foot or hand can result in symptoms such as pain, numbness, and sensory loss. In later stages, this neuropathy may progress to affect the ankle and calf, ultimately leading to ulcerative disability or even amputation, significantly impacting the patient's quality of life [5, 6]. Although insulin therapy is effective in regulating blood glucose in diabetic patients, there are still many patients who will gradually develop DPN-related symptoms with the development of DM [7, 8]. In addition, though pharmacological interventions such as anticonvulsants, aldose reductase inhibitors, and antidepressants could improve DPN, they are also associated with common adverse effects [9, 10]. These adverse effects, including nausea, dizziness, weight gain, and dry mouth, occur in nearly half of the population taking the medication and exhibit dose-dependent characteristics [9]. Therefore, there is a critical need to develop effective treatments for DPN.

An increasing body of research has demonstrated the association between DPN and various factors, including hyperglycemia, endoplasmic reticulum (ER) stress, oxidative stress, viral infections, inflammatory cytokines, gene mutations, apoptosis, and autophagy [11-14]. Among these factors, ER stress has emerged as a closely linked factor to the progression of DPN [15, 16]. ER stress in hyperglycemia can lead to dysregulation of transcription and gene expression, disruption of cellular signaling pathways, ion channel dysfunction, and metabolic disorders [17, 18]. Simultaneously, ER stress also exacerbates oxidative stress, triggering an inflammatory response that promotes apoptosis, ultimately leading to widespread damage to neural tissue [19, 20]. The RNA-dependent protein kinase (PKR)-like ER kinase (PERK)-C/EBP homologous protein (CHOP) is one of the classical pathways of ER stress [21]. As well as PERK, as a key factor in the response to ER stress, is activated through the dissociation of GRP78 under ER stress conditions. When an excess of unfolded proteins accumulates in the ER, GRP78 dissociates from PERK, promoting its dimerization and autophosphorylation, thereby activating its kinase activity. Once activated, PERK phosphorylates eIF2α, leading to global translation inhibition while simultaneously promoting selective translation of ATF4. This results in sustained overexpression of ATF4, which in turn initiates the stress response [20, 22]. Which subsequently transduces to the downstream CHOP, an important molecule involved in the transition from anti-apoptosis to pro-apoptosis during ER stress, ultimately leading to cell failure [23]. In a rodent model of DM, the administration of an ER stress inhibitor reduces ER stress by restoring ER function and improves neuronal functional phenotypes, such as nerve conduction velocities and behavioral responses to mechanical and thermal stimuli [24]. Therefore, targeting the improvement of the PERK/ATF4/CHOP pathway for the treatment of DPN may be a promising therapeutic strategy.

Traditional Chinese medicine (TCM) shows promise in the treatment of DPN, particularly in cases of polyneuropathy [25-27]. Research data suggested that commonly used herbs with neuroprotective, antioxidant, and anti-neuroinflammatory effects may improve the progression of DPN [28, 29]. Traditional medicine research has shown that in diabetes, prolonged Xiao Ke syndrome can lead to blockage, stasis, and obstruction of the meridians, which may result in the development of DPN. As the condition progresses, the imbalance of Qi in the meridians induces a series of clinical symptoms. The Qizhi Kebitong Formula (QKF) was the in-hospital preparation of the Affiliated Hospital of Changchun University of Traditional Chinese Medicine, whose pharmaceutical composition includes *Astragalus membranaceus* (Fisch.) Bunge (Huang-qi), *Ramulus mori* the twig of *Morus alba* L. (Sang-zhi), *Siegesbeckiae herba* (Xi-xian-cao), *Clematis chinensis* Osbeck (Wei-ling-xian), caulis of *Spatholobus suberectus* Dunn (Ji-xue-teng), *Achyranthes bidentata* Blume (Niu-xi), *Scorpio* (Quan-xie). It has been used clinically for more than 20 years as an effective formula for the treatment of DPN [30]. QKF is effective in tonifying Qi, invigorating blood circulation and resolving blood stasis, clinically relieving patients from numbness of hands and feet, and body pain [30]. However, the underlying molecular mechanism is unclear.

In this study, we established a streptozotocin (STZ)-induced mouse model of DPN through intraperitoneal injection of STZ to evaluate the therapeutic effect of QKF and conducted a series of experimental validations to explore the underlying mechanism by which QKF alleviates the DPN model. Our findings revealed that QKF ameliorated STZ-induced diabetes mice sciatic nerve (SN) injury and provided preliminary insights into the underlying molecular mechanism, which may be related to the inhibition of PERK/ATF4/CHOP and subsequent reduction of apoptosis.

## 2 MATERIALS AND METHODS

### 2.1 HPLC/QTOF-MS Raw Materials and Sample Preparation

QKF is made up of seven herbs, including Huang-qi, Sang-zhi, Xi-xian-cao, Wei-ling-xian, Ji-xue-teng, Niu-xi, and Quan-xie. The herbal dosage ratio used was 6:4:4:3:3:2:1. According to the standard procedures, the seven mentioned medicines were decocted in 1000 mL of distilled water at 100°C until a 100 mL extract of QKF was obtained [31]. The obtained extract was cooled, followed by centrifugation at 8,000 rpm for 10 minutes to collect the supernatant. The QKF extract was subsequently freeze-dried to obtain a powdery form, which was then diluted to a concentration of 5 mg/mL using ultrapure water. The resulting solution was subjected to centrifugation again at 14,000 rpm for 10 minutes at 4°C, and the supernatant was collected for further analysis.

We used a total of 15 standards in this experiment: mulberroside F; calycosin 7-o-glucoside (ST08820120-5240); ononin (3811); methylnissolin; methylnissolin-3-o-glucoside (ST19060210); isomucronulatol 7-o-glucoside; astragaloside III (DST181118-018); cyclocephaloside II; astragaloside II (DST180315-023); astragaloside I (DST190216-016); isoastragaloside I (DST180315-022); genistin (9435); betulonicacid (DST190508-026); isoastragaloside IV (ST81100105); astragaloside IV (DST190711-015).

A sample extract of 100 mg from each of the 10 batches of different sources of QKF was accurately weighed and dissolved in 1 mL of a 30% methanol solution. The resulting solution was vortexed to prepare a 100 mg/mL test material solution for fingerprinting and similarity evaluation.

### 2.2 UHPLC/QTOF-MS Analytical Method

The instrument was a Waters Xevo G2-XS QTof high-resolution mass spectrometer (Waters Corporation, USA). The MS^E^ acquisition method was used for mass spectrometry detection, and data were acquired separately for positive and negative ion scanning modes. MS^E^ data were calibrated and processed using UNIFI^TM^ 1.9.3.0 software (Waters Corporation, USA). QKF fingerprinting and similarity evaluation using Waters Acquity Arc high-performance liquid chromatography (HPLC). The HPLC fingerprints of 10 batches of QKF samples were analyzed and processed by using the “Traditional Chinese Medicine Chromatographic Fingerprint Evaluation System, 2004 Edition”.

### 2.3 Animal Experimental Design

Eight-week-old male C57BL/6 mice weighing 19-21 g were purchased from Changchun Yisi Experimental Animal Technology Co., Ltd. [SCXK (Ji) 2023-0002, Changchun, Jilin]. The feeding and use of all animals comply with the guiding principles of the Animal Care and Use Committee of the Institute of Nutritional Science (Ethics Approval No: 2022475). The mice were provided with ad libitum access to water and food and were housed under standard experimental conditions (12-hour light-dark cycle, 21-24°C). The C57BL/6 mice with DPN were prepared using a previously published method [32]. In summary, C57BL/6 male mice were fed a normal diet for one week, followed by a single intraperitoneal injection of a dose of 120 mg/kg STZ (Sigma-Aldrich). A total of 12 mice were selected as the control (ctrl) group and injected with an equivalent volume of citrate buffer (pH 4.5). After 72 hours of injection, mice with random blood glucose levels ≥ 16.7 mmol/l were identified as T1DM mice. All T1DM mice were randomly divided into 5 groups, with 12 mice in each group. The grouping is as follows: STZ group: pre-established DPN mice treated with 0.2 ml of 0.9% saline by gavage; QKF groups: pre-established DPN mice treated with QKF (2 g/kg/day, 4 g/kg/day, 8 g/kg/day); MBT (mecobalamin tablets) group: pre-established DPN mice treated with MBT (189 mg/kg). Drug dose conversions for animal experiments were based on the Chinese Pharmacopoeia (2020 edition).

### 2.4 Blood Glucose and Weight Measurement

To confirm the diabetic state of the mice, random blood glucose levels and body weight were measured every two weeks. Blood glucose measurements were obtained by collecting blood from the tail vein using a Roche blood glucose meter (Roche, Germany).

### 2.5 Behavioral Tests

The mice were placed on a heating plate maintained at a temperature of 55°C, and the latency for paw licking or jumping behavior was recorded. If there was no response within 30 seconds, the latency was recorded as 30 seconds. Additionally, the tail flick test was conducted by exposing the mouse’s tail to a light source and recording the time taken for the tail to flap or retract. All experimental procedures involving the mice began after a 30-minute acclimatization period in the experimental area. Individual measurements were then taken at least 3 times with a minimum interval of 20 minutes, and the average value was considered as the final data.

### 2.6 Nerve Conduction Velocity Test

The nerve conduction velocity in mice was assessed based on previously published methods [33, 34]. Bipolar electrodes were used to measure the sensory nerve conduction velocity (SNCV) and motor nerve conduction velocity (MNCV), as well as the amplitude of the SN. Nerve conduction velocity (cm/s) was calculated as the distance between the two recording electrodes (in cm) divided by the difference in latency of the compound potential between the two channels (in seconds).

### 2.7 Regional Blood Flow Perfusion Measurement

Laser speckle contrast imaging (LSCI) is a novel and effective method for monitoring and imaging blood flow, offering technical advantages such as non-contact, high-resolution, and rapid full-field imaging. This non-invasive technique could detect perineural vascular blood flow. The groups underwent LSCI 12 weeks after administration, with the distance from the camera to the target area set at 10-15 cm. In each blood flow image, regions of interest (ROI) were selected, and the average ROI value of the region was calculated to analyze the blood flow data.

### 2.8 Morphological Observation of SN

Morphologic changes in the SN were assessed using hematoxylin and eosin (H&E) staining, Nissl staining, glycine silver staining and luxol fast blue (LFB) staining.

#### 2.8.1 H&E Staining

After 12 weeks of administration, mice were euthanized by intraperitoneal injection of 1% pentobarbital sodium. The SN were collected aseptically, fixed in 4% paraformaldehyde for 24 hours, and subsequently dehydrated using a series of different grades of alcohol. Following treatment with xylene, the specimens were embedded in paraffin blocks, transversely and serially sectioned, and stained using the H&E staining kit (Servicebio, G1003). Photographic analysis was performed using an orthogonal light microscope (Nikon Eclipse E100, Japan).

#### 2.8.2 Nissl Staining

The sections were placed in Nissl’s stain solution (Servicebio, G1036) for 2-5 minutes, lightly rinsed with distilled water, and slightly differentiated using 0.1% acetic acid. After completing slide imaging, the number of neurons within three 400x fields of view on each slide, as well as the corresponding tissue area, were measured using Image-Pro Plus 6.0 analysis software, with millimeters as the standard unit. Neuronal density was calculated by dividing the number of neurons by the tissue area.

#### 2.8.3 Glycine Silver Staining

The sections were sequentially immersed in xylene I, xylene II, anhydrous ethanol I, anhydrous ethanol II, and 75% ethanol. Subsequently, they were individually immersed in silver staining solutions C, B, and A containing glycine, followed by rinsing with distilled water, dehydration, mounting the slides, and conducting image capture. Quantitative analysis was performed using the Area Quantification v2.1.3 module of Halo v3.0.311.314 analysis software (Indica Labs).

#### 2.8.4 LFB Staining

The Myelin Staining Kit (Servicebio, G1030) was used for staining. Myelin Staining Solution A was preheated in a 60°C oven for 30 minutes, and the sections were immersed in the solution for 1 hour. After removing and quickly rinsing the sections, they were briefly differentiated in Myelin Staining Solution B for 2 seconds (while still hot), and then directly immersed in Myelin Staining Solution C for 15 seconds until the myelin turned a blue background. Following restaining with eosin, the sections were dehydrated and sealed, and images were captured and analyzed using Halo v3.0.311.314 analysis software.

### 2.9 Ultrastructural Observations of the SN

The SN was sampled and placed into a petri dish containing electron microscope fixative (Servicebio, G1102). The nerve was cut into 1 mm^3^ pieces of tissue, which were then placed into an Eppendorf tube containing electron microscope fixative and fixed further at 4°C. The tissue was dehydrated and embedded at room temperature, and 60-80 nm ultra-thin sections were prepared. At least two randomly selected photographs from each sample were used to quantify axonal degeneration using a transmission electron microscope (HITACHI, HT7800). The g-ratio was calculated by dividing the measured axon diameter by the diameter of the myelinated fibers [35].

### 2.10 Real-time Quantitative PCR (RT-qPCR) Analysis

Total RNA samples were isolated from cells using the Magnetic Bead Method RNA Extraction Kit (Tiangen Biotech, Beijing). Use cDNA synthesis premix reagent (Tiangen Biotech, Beijing) to transcribe complementary DNA (cDNA) from the same concentration of total RNA product. After quantification, relative gene expression analysis was performed using the 2^-ΔΔCT^ method [36]. The sequence of primers used in the experiment is shown in Table **1**.

### 2.11 Conventional Immunohistochemistry

After deparaffinization of the paraffin sections to water, antigen retrieval was performed using citrate antigen retrieval buffer (pH 6.0) (Servicebio, G1202). Subsequently, endogenous peroxidase activity was quenched, and non-specific binding was blocked by the addition of goat serum. Primary antibodies were incubated overnight at 4°C using the indicated dilutions. Sections were then rinsed and incubated with the corresponding species-specific secondary antibody at 37°C for 50 minutes. The nuclei were restained by adding DAB color solution (Servicebio, G1212), and the results were subsequently interpreted. Immunohistochemistry analysis was performed using Aipathwell^®^ software (Servicebio^®^).

### 2.12 Western Blot Analysis

The protein concentration was assayed by lysing the SN in RIPA buffer (Genbiotech) for 30 minutes. Subsequently, the protein (30 μg) was boiled at 100°C for 5 minutes and subjected to 8%, 10%, or 12% SDS-PAGE gel electrophoresis. The separated proteins were then transferred onto a PVDF membrane. Primary antibody incubation was performed using the following antibodies: anti-p-PERK (1:1000, Affinity Biosciences), anti-PERK (1:500, Proteintech), anti-GRP78 (1:1000, Proteintech), anti-ATF4 (1:1000, Proteintech), anti-p-eIF2a (1:1000, Abways Technology), anti-eIF2a (1:5000, Proteintech), anti-CHOP (1:1000, Proteintech), anti-Bcl-2 (1:2000, Proteintech), anti-Bax (1:5000, Proteintech), and anti-β-actin (1:10000, Proteintech). After incubation with the secondary antibody, the target bands were photographed, and ImageJ software was used to analyze the optical density values of the target bands.

### 2.13 Statistical Analysis

Statistical analysis was performed using GraphPad Prism v8.0 software. Data were expressed as mean ± SEM. Differences between groups were compared by one-way analysis of variance (ANOVA) with post hoc test of Least Significance Difference (LSD). Significance was set at *p* < 0.05.

## 3 RESULTS

### 3.1 Chemical Profiling of QKF

**Fig. (1):**
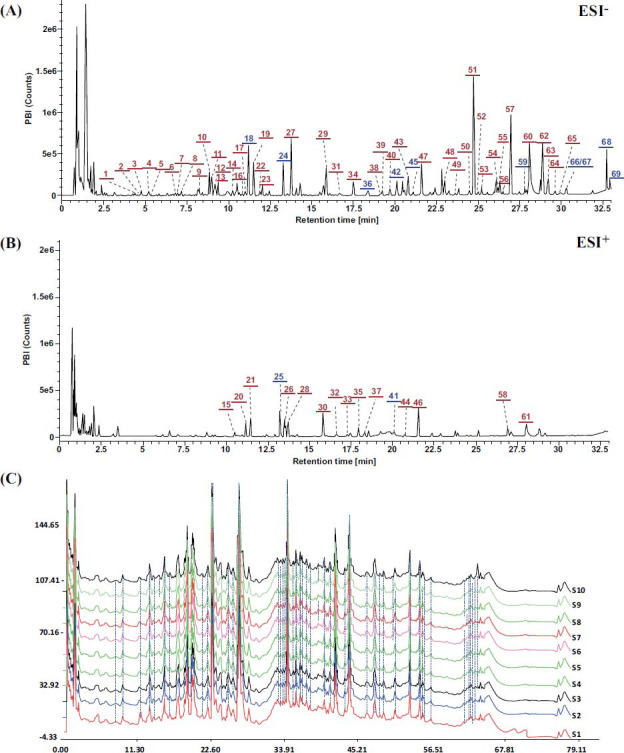
UHPLC/QTOF-MS of QKF. (**A**) Negative ion scanning modes. (**B**) Positive ion scanning modes. (**C**) Fingerprints of 10 batches of samples.

Base peak ion chromatograms were obtained in the positive and negative ion modes, as shown in Figs. (**1A**, **B**). A total of 69 compounds were identified or deduced, of which 12 components were confirmed by comparison with reference substances. Fourteen compounds were identified or deduced from the single herb Huang-qi, primarily including astragaloside I, astragaloside II, and astragaloside III, along with several others. For Ji-xue-teng, 14 compounds were identified or deduced, primarily consisting of gallocatechin, asperulosidic acid, and primeverin. For Niu-xi, 11 compounds were identified or deduced, such as lentztrehalose, hythiemoside A. From Sang-zhi, four compounds were identified or deduced, including mulberroside A and mulberroside F. The identification or deduction of 18 compounds from Wei-ling-xian was achieved, with major components being 5-galloylshikimic acid, catechin hydrate, genistin, and others. Lastly, 8 compounds were identified or deduced from Xi-xian-cao, including hypsiziprenol-AA14, Ent-17-hydroxy-16βH-kauran-19-oic acid, and others. Detailed identification information for the 69 compounds can be found in Table **S1**. The fingerprint patterns of 10 batches of QKF were compared with the generated reference chromatograms (Fig. **1C**). The similarity values ranged from 0.932 to 0.980, all exceeding 0.90. The results indicated minimal overall quality variation among the 10 batches of QKF.

### 3.2 QKF Improved Neurological Functions Associated with DPN Mice

**Fig. (2):**
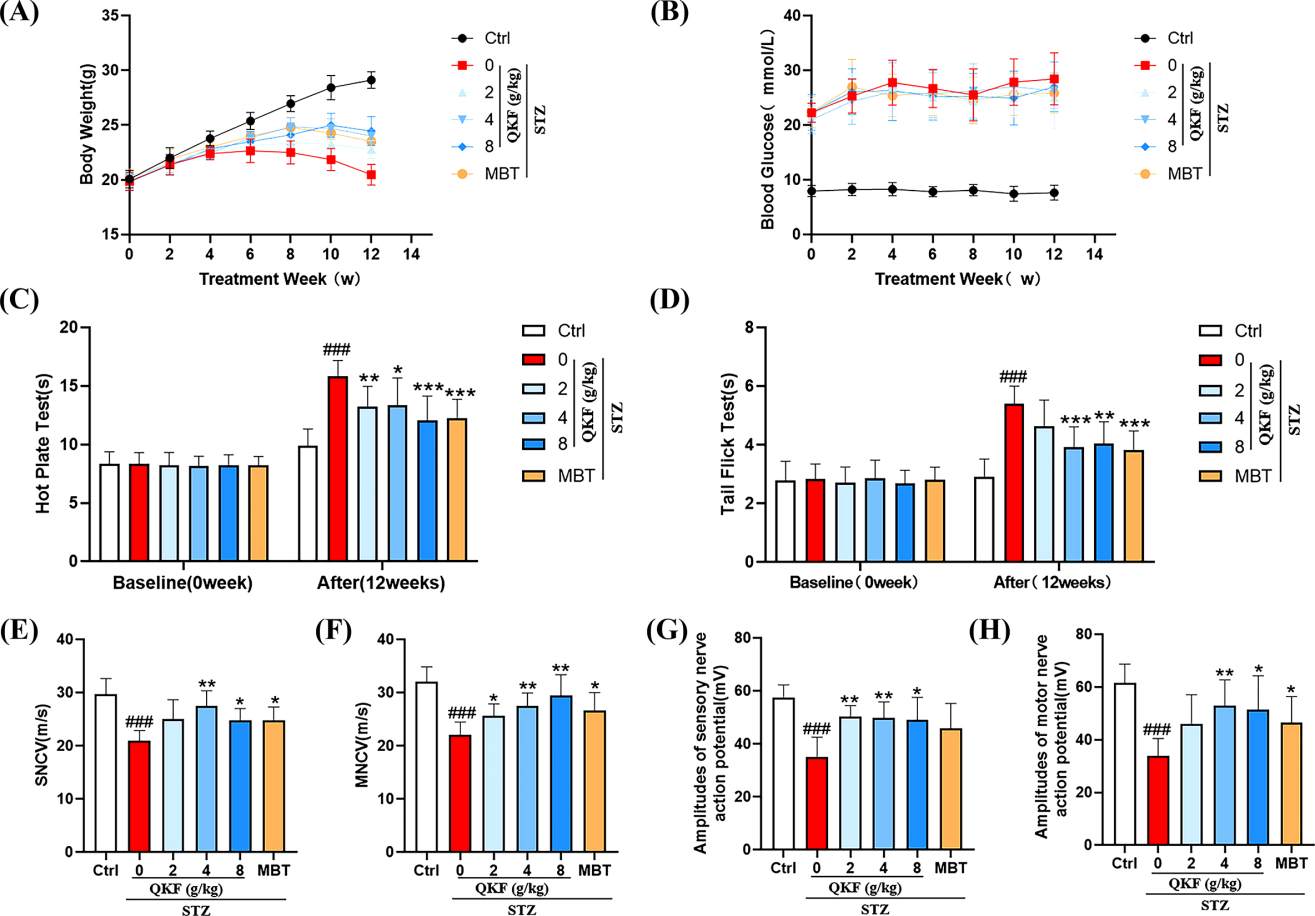
QKF improved DPN-related phenotypic characteristics and neurological functions in mice. (**A**) Body weight. (**B**) Blood glucose. (**C**) Hot Plate Test. (**D**) Tail Flick Test. (**E**) SNCV: sensory nerve conduction velocity. (**F**) MNCV: motor nerve conduction velocity. (**G**) Amplitudes of sensory nerve action potential. (**H**) Amplitudes of motor nerve action potential. Data were presented as mean ± SEM (n=8 for A-D; n=5 for E-H). ^#^, *versus*. Ctrl group, ^#^*p* < 0.05, ^##^*p* < 0.01, ^###^*p* < 0.001; *, *versus*. STZ group, *p < 0.05, **p < 0.01, ***p < 0.001.

Considering the demonstrated potential of QKF in clinical treatment, we systematically investigated its role in ameliorating the phenotype and neurological aspects of DPN mice. Fig. (**2A**) showed that the mice in the ctrl group continued to gain weight as the experimental period was prolonged, whereas the mice in the STZ group gradually lost weight after 6 weeks of the experiment. The body weights of QKF (4 g/kg) and MBT groups were elevated (23.83 ± 0.52, 23.96 ± 1.05) and statistically significant when compared with those of mice in the STZ group (22.64 ± 1.09) after 6 weeks of treatment (Table **2**, *p* < 0.05). The results in Fig. (**2B**) suggested that the therapeutic effect of QKF may not be due to a role in lowering blood glucose, as there was a reduction in blood glucose in the QKF as well as MBT groups, but it was not statistically significant. Figs. (**2C**, **D**) showed that STZ group exhibited typical DPN phenotypes after 12 weeks, including up-regulation of perception levels and prolonged thermal latency, whereas both QKF and MBT improved these manifestations after 12 weeks of treatment. Nerve conduction velocity measurement is considered the gold standard for DPN [37]. Revealed significant decreases in SNCV, MNCV, amplitudes of sensory nerve action potential, and amplitudes of motor nerve action potential in the STZ group compared with the ctrl group (Figs. **2E**-**H**), suggest the presence of neuropathy in STZ-induce mice after 12 weeks of the experiment. QKF effectively prevented or alleviated all the above indicators of neurological disorders in DPN mice (*p* < 0.05). These findings indicate that QKF has the potential to enhance neurological function in STZ-induced diabetes mellitus mice.

### 3.3 QKF Improves Peripheral Blood Flow in DPN Mice

**Fig. (3):**
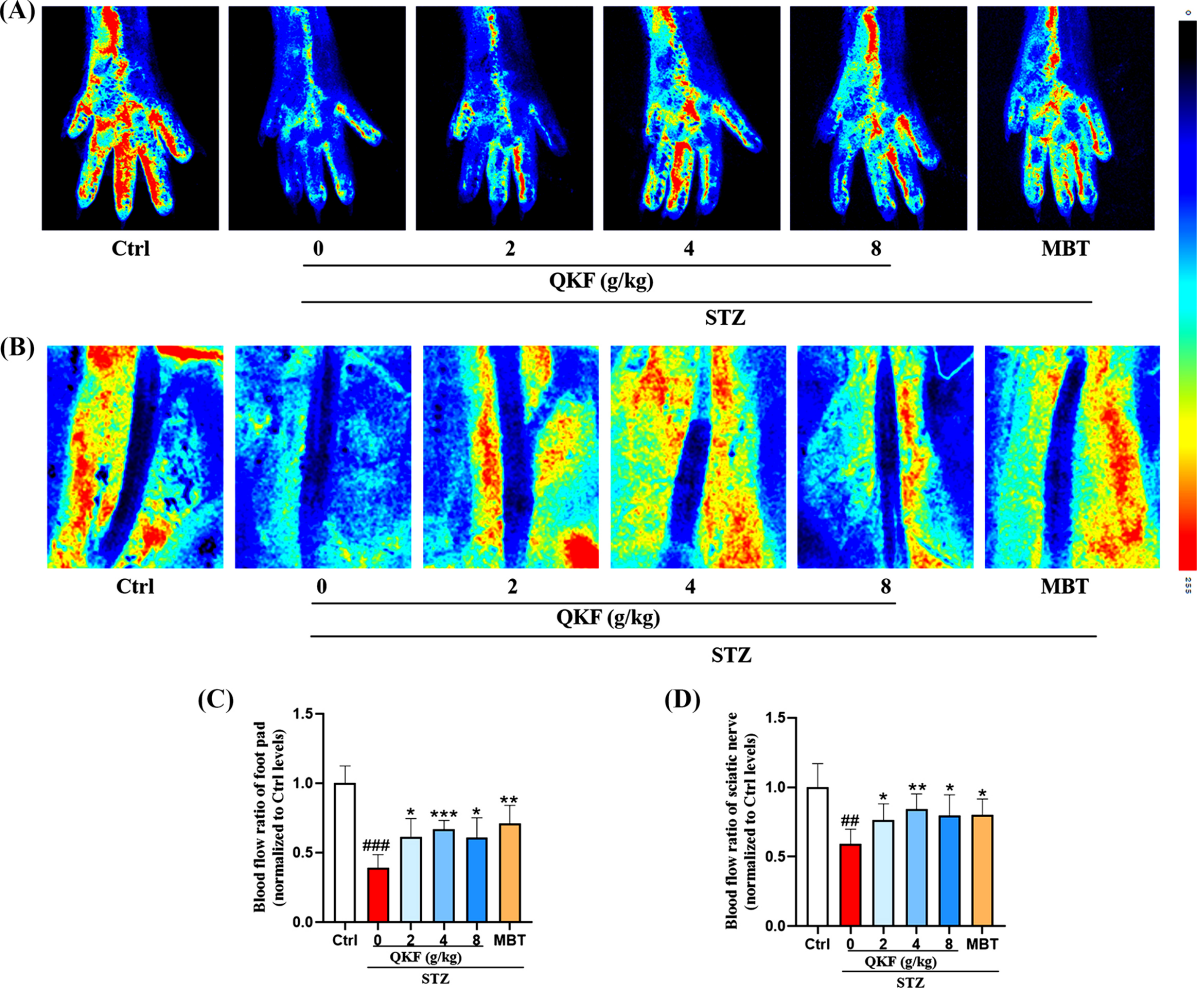
QKF improves peripheral blood flow in DPN mice. (**A**, **B**) Representative images of blood perfusion volume in the footpad tissue and sciatic nerve region of mice in the ctrl, STZ, QKF, and MBT groups. (**C**, **D**; n=5) Quantification of blood perfusion normalized to the ctrl group. The lowest blood flow is represented by blue, the highest blood flow is represented by red, and intermediate levels are represented by green and yellow. ^#^, *versus*. ctrl group, ^#^*p* < 0.05, ^##^*p* < 0.01, ^###^*p* < 0.001; *, *versus*. STZ group, * *p* < 0.05, **p < 0.01, ***p < 0.001.

To investigate whether QKF treatment could improve neurovascular function, we used LSCI to detect a significant decrease in blood perfusion around the foot pads and SN in the STZ group compared to the ctrl group (Fig. **3**, *p* < 0.01). However, after 12 weeks of treatment with QKF and MBT, there was an increase in blood perfusion. These results indicate that QKF improved peripheral vascular function and increased perfusion in DPN mice.

### 3.4 QKF Ameliorated Pathologic SN Injury in DPN Mice

**Fig. (4):**
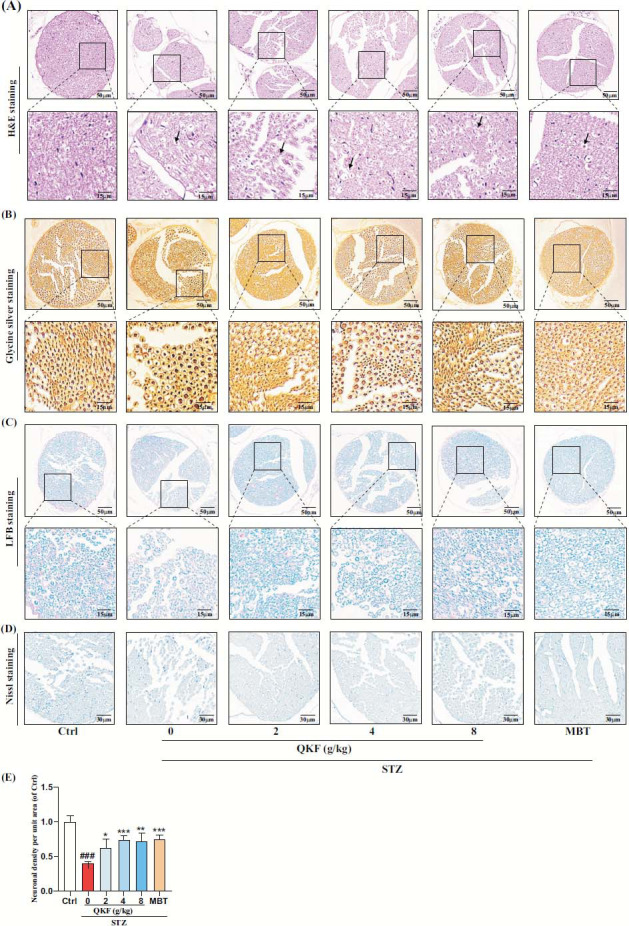
H&E, Glycine silver, LFB and Nissl staining for observing pathologic changes of sciatic nerve tissues. (**A**) H&E staining (scale bars 50 μm and 15 μm, respectively). (**B**) Glycine silver staining (scale bars 50 μm and 15 μm, respectively). (**C**) LFB staining (scale bars 50 μm and 15 μm, respectively). (**D**) Nissl staining (scale bar 30 μm). (**E**) Quantification of neuronal density per unit area in Nissl staining, N=3 per group. ^#^, *versus*. ctrl group, ^#^ *p* < 0.05, ^##^ *p* < 0.01, ^###^ *p* < 0.001; *, *versus*. STZ group. *p < 0.05, **p < 0.01, ***p < 0.001. ^#^*p* <0.05; ^##^*p* <0.01.

To further assess the protective potential of QKF against STZ-induced neuropathy in mice, we conducted a series of staining techniques, including H&E, glycine silver, LFB and nissl staining. According to the findings depicted in Fig. (**4A**), the ctrl mice exhibited intact morphology and well-organized structure of SN fibers, with myelinated nerve fibers displaying a systematic arrangement. In the STZ group, more nerve fibers were swollen (black arrowheads) and vacuolated, local structural disorganization was seen, the nerve fibers were poorly demarcated. The results of glycine silver staining indicated that in the STZ group, SN fibers were disorganized, and axons were swollen, vacuolated and degenerated (Fig. **4B**). LFB staining showed that the SN myelin sheath in the STZ group was darker in coloration and showed changes such as swelling, curving into a spherical shape, and fracture (Fig. **4C**). Mice treated with QKF and MBT exhibited less severe pathological changes compared to those in the STZ group, as mentioned earlier. Nissl staining showed that compared with the ctrl group, the STZ group had a reduced number of neurons, structural changes, and a lower density of neurons per unit area. Meanwhile, the QKF and MBT groups showed significant improvement in neuronal morphology and structure and increased neuronal density per unit area compared with the STZ group (Figs. **4D**, **E**, *p* < 0.05). Through comparative analysis of various histological staining, it has been demonstrated that QKF can improve the morphological and structural defects of the sciatic nerve induced by DPN, further confirming the potential of QKF in treating DPN-associated sciatic neuropathy.

### 3.5 QKF Improved SN Ultrastructure in DPN Mice

**Fig. (5):**
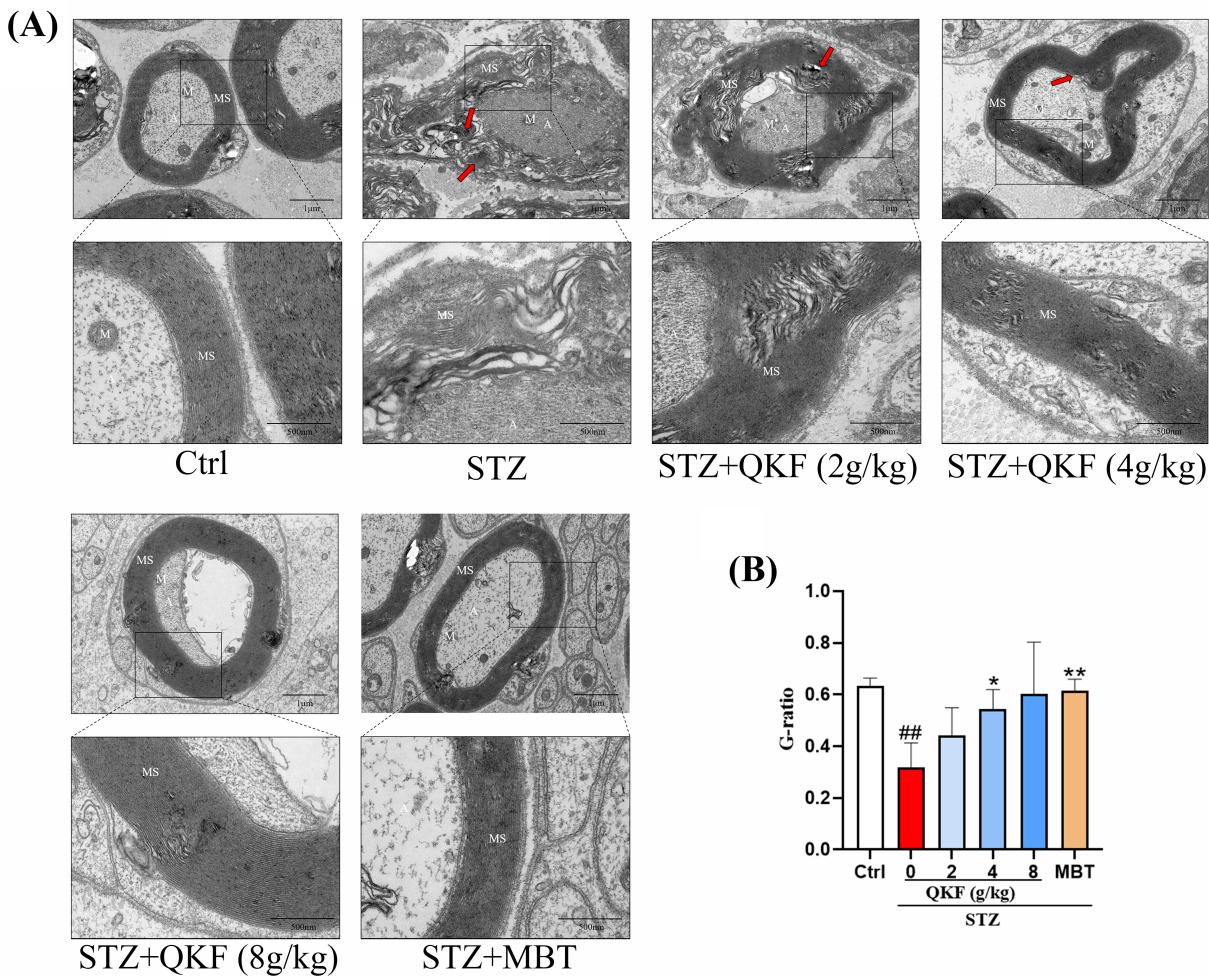
Transmission electron microscopy observation of the ultrastructure of the sciatic nerve. (**A**) Representative electron microscopy images (Scale bars are 1 μm and 500 nm, respectively). (**B**) g-ratio. Presented as mean ± SEM (n=3). ^#^, *versus*. Ctrl group, ^#^*p* < 0.05, ^##^*p* < 0.01; *, *versus*. STZ group, *p < 0.05, **p < 0.01.

To further analyze QKF's therapeutic effect on SN injury, we observed the ultrastructure of the SN using transmission electron microscopy (TEM). Mice in the STZ group exhibited severe damage to the myelinated nerves compared to the ctrl group, with irregularly shaped myelin sheaths (MS), demyelination, delamination, loose and disorganized structural arrangement, and extensive lysis and degeneration (red arrows). The axon (A) was markedly atrophied, and the stroma was slightly condensed. Mitochondrial (M) membrane rupture, cristae fracture lysis, matrix lysis, and partial vacuolar transformation were also observed. Schwann cells at the outer edge were extensively damaged and disintegrated (Fig. **5A**). QKF or MBT treatment significantly attenuated these phenotypes. Additionally, the g-ratio, which reflects the ability of nerve conduction and is the ratio of axon diameter to fiber diameter of myelinated nerve fibers, was significantly altered in the STZ group compared to the ctrl group, but QKF (4 g/kg) and MBT may have reversed these changes (Fig. **5B**). According to the findings, it was observed that STZ-induced neurotoxic defects in the SN, including axonal degeneration and myelin damage, were significantly rescued by QKF.

### 3.6 QKF Regulated ER Stress and Apoptosis-related mRNA Expression in Mouse SN

**Fig. (6):**
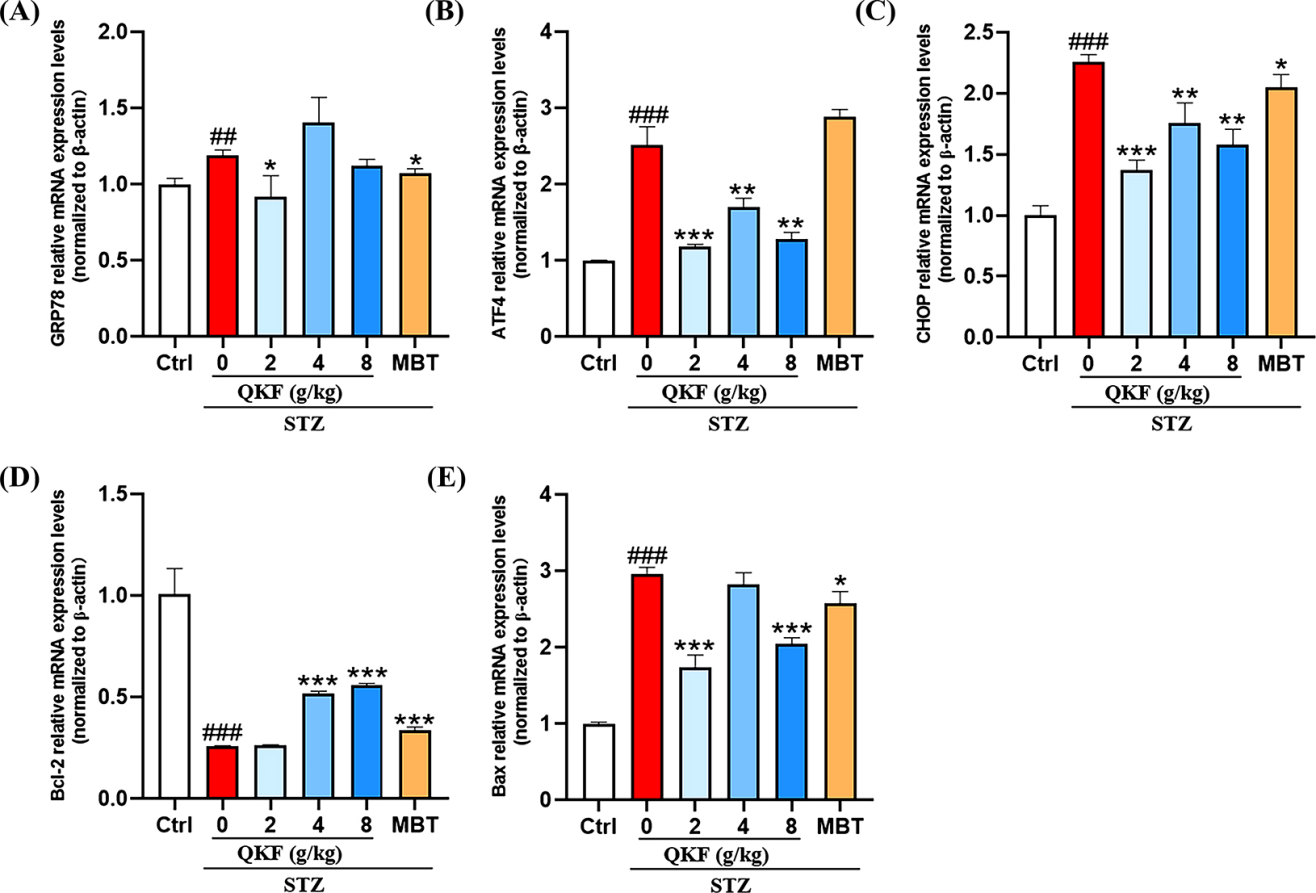
QKF improves ER stress and apoptosis-related mRNA expression in sciatic nerve of DPN mice. (**A-E**) The relative mRNA expression levels of GRP78, ATF4, CHOP, Bcl-2 and Bax were calculated and normalized to β-actin levels. ^#^, *versus*. Ctrl group, ^#^*p* < 0.05, ^##^*p* < 0.01, ^###^*p* < 0.001; *, *versus*. STZ group, *p < 0.05, **p < 0.01, ***p < 0.001.

To investigate whether ER stress was involved in the regulation of DPN alleviation by QKF, we used RT-qPCR to analyze the mRNA expression levels of relevant key markers in mouse SN. Our findings revealed that the mRNA expression levels of ER stress genes GRP78, ATF4, and CHOP in the STZ group were elevated compared to the ctrl group, indicating the occurrence of ER stress. After QKF and MBT treatments, the mRNA expression of these genes showed a significant reduction (Figs. **6A**-**C**, *p* < 0.05), indicating that QKF downregulated these genes mRNA expression and can effectively ameliorate ER stress. In addition, CHOP can trigger apoptosis by regulating the Bcl-2 protein family. Consequently, we assessed the mRNA expression levels of Bcl-2 and Bax. Bcl-2 and Bax were crucial regulators of apoptosis and were part of the final common pathway in apoptosis regulation [38, 39]. In comparison to the ctrl group, the STZ group mice exhibited a decrease in the expression of the Bcl-2while the expression of the pro-apoptosis-related gene Bax was elevated (*p* < 0.05, Figs. **6D**, **E**). The results suggested that QKF can alleviate ER stress and lower the expression of mRNA related to apoptosis in the SN of mice with DPN.

### 3.7 QKF Suppressed ER Stress and Apoptosis-related Protein Expression in DPN Mice through the PERK/ATF4/CHOP Signaling Pathway

**Fig. (7):**
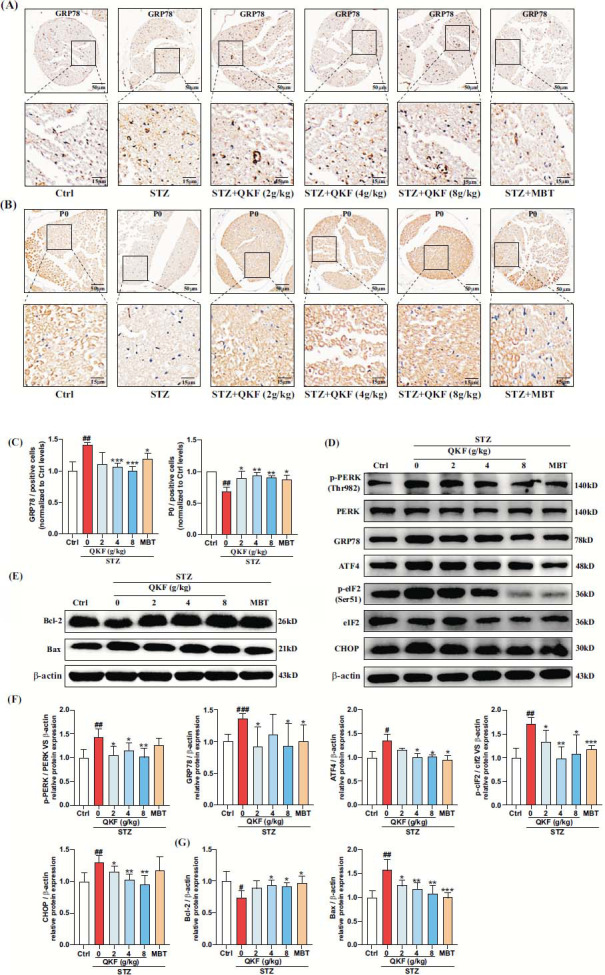
The levels of PERK/ATF4/CHOP ER stress signaling pathway in STZ-induced mice. (**A**, **B**) Representative images depicting changes in GRP78 and P0 expression levels through immunohistochemical analysis (Scale bars are 50 μm and 15 μm, respectively). (**C**) Quantitative analysis of GRP78 and P0-positive cells (n=3), normalized compared to the Ctrl group. (**D**) Representative graphs showing the expression levels of ER stress-associated proteins, including p-PERK, PERK, GRP78, ATF4, p-eIF2, eIF2, and CHOP. (**E**) Representative graph displaying the expression levels of apoptosis-related proteins, Bcl-2 and Bax. (**F**, **G**) Quantitative plots of protein expression levels normalized to β-actin. ^#^, *versus*. Ctrl group, ^#^*p* < 0.05, ^##^*p* < 0.01, ^###^*p* < 0.001; *, *versus*. STZ group, *p < 0.05, **p < 0.01, ***p < 0.001.

Subsequently, we conducted immunohistochemistry and Western blot analysis. Immunohistochemical results revealed an elevated ratio of GRP78-positive cells in the SN of mice in the STZ group compared to the ctrl group (Figs. **7A**, **C**, *p* < 0.05). Both the QKF and MBT groups exhibited a reduction in the ratio of GRP78-positive cells compared with the STZ group. In peripheral nerve myelin, P0 accounts for about 50% of all proteins and is a key marker protein for the functional expression of peripheral nerve myelin [40, 41]. Furthermore, as shown in Figs. (**7B**, **C**), the ratio of P0-positive cells in the SN of mice in the STZ group was decreased compared to the ctrl group (*p* < 0.05). The QKF and MBT groups elevated the ratio of P0-positive cells in the mouse SN and improved myelin function compared with the STZ group. The Western blot results demonstrated significant elevation in the protein expression levels of p-PERK/PERK, GRP78, p-eIF2/eIF2, ATF4, and CHOP in the SN of mice in the STZ group (Figs. **7D**, **F**, *p* < 0.05), indicating the presence of ER stress in these mice. However, after 12 weeks of QKF or MBT interventions, these changes were ameliorated. Further investigations revealed a down-regulation of the anti-apoptotic protein Bcl-2 and an up-regulation of the pro-apoptotic protein Bax in the SN tissues of mice in the STZ group, suggesting an increased level of apoptosis. Remarkably, QKF inhibited apoptosis in the SN tissues of DPN mice by up-regulating Bcl-2 and down-regulating Bax (Figs. **7E**, **G**, *p* < 0.05). Taken together, these results indicated that the regulation of the PERK/ATF4/CHOP ER stress pathway may be involved in the protective effects exhibited of QKF against DPN in mouse model.

## 4 DISCUSSION

TCM, as a significant branch of medicine, offers unique insights and therapeutic effects on human health and treatment approaches. In our study, we established a DPN mouse model to further investigate the therapeutic effects of QKF on SN function and the improvement of nerve fiber myelin pathology. Additionally, we observed changes in protein expression in the SN of DPN mice after QKF treatment, which were found to be associated with ER stress and cellular apoptosis. These findings suggest that QKF has a protective effect on nerve fibers and the morphology and function of the ER in DPN. However, traditional herbal medicine is a highly complex chemical system that contains numerous metabolites with complex structures, similar properties, and unstable components. HPLC/QTOF-MS is widely used for the identification and analysis of complex components in traditional herbal medicine. Therefore, we analyzed the chemical composition of QKF using HPLC/QTOF-MS and identified or deduced a total of 69 compounds. These compounds primarily include biosaponins, terpenoids, ketones, *etc*. These components may contribute to the pharmacological effects of QKF, producing significant neuroprotective effects. For example, previous research has demonstrated that Isoastragaloside I, a natural saponin constituent found in *Astragalus membranaceus*, exhibits neuroprotective, immunomodulatory, and antioxidative properties [42]. Furthermore, research suggests that Mulberroside F isolated from *Ramulus mori* may exert neuroprotective effects by inhibiting tyrosinase activity [43, 44]. Subsequent investigations will focus on elucidating the precise mechanisms through which these ingredients exert their regulatory effects on DPN.

The C57BL/6 mice were administered STZ to induce DPN, thereby establishing an animal model extensively utilized in research on diabetic sequelae. As the experimental duration extended, mice in the Control group consistently exhibited weight gain. In contrast, the mice in the STZ group began to manifest weight loss after the 6-week mark. This trend implies that in the initial phase of the pathology, homeostatic regulation might be maintained *via* compensatory biological processes. However, persistent hyperglycemia in the intermediate and advanced stages precipitates energy depletion and disrupts the physiological metabolism of lipids, proteins, and carbohydrates. The statistically significant comparison of body weights of mice in the QKF and DM groups after 6 weeks of treatment suggests that perhaps QKF could counteract the negative effects of DM to some extent, thereby delaying the onset of weight loss. In addition, our findings suggested that the therapeutic effect of QKF may not be due to the lowering of blood glucose. There was a decrease in blood glucose in the QKF as well as MBT group compared to the DM group, but it was not statistically significant (*p* > 0.05). A persistent hyperglycemic state can lead to ER stress [45, 46]. We hypothesize that the therapeutic effect of QKF may involve the preservation of neurovascular function by attenuating ER stress and inhibiting apoptosis, thereby slowing the progression of DPN. In addition, DPN is often associated with hyperalgesia and loss of sensation [47]. The present study showed that DM mice exhibited a typical DPN phenotype after 12 weeks of experimentation, including up-regulation of perception levels, prolonged thermal latency, and reduced nerve conduction velocity, as confirmed by several recent basic studies [32, 48]. In contrast, both QKF and MBT reversed the above indicators after 12 weeks of treatment, suggesting that QKF has a therapeutic or preventive effect on DPN. DPN is usually characterized by loss of myelin integrity and apoptosis of Schwann cells. QKF showed reparative/protective effects on the SN, ameliorated demyelination and axonal damage, and improved neurovascular function. It is worth reflecting, however, that our observations are based only on the mouse model and may not fully reproduce the complex DPN pathology in humans.

To confirm the role of ER stress in DPN disease progression, we detected GRP78, a key regulatory protein that plays an important role in ER stress [49]. Under physiological conditions, GRP78 expression levels are low and restricted to ER transmembrane receptors. However, under pathological conditions such as hypoxia, ischemia and high-glycemic environments, ER stress will be activated and unfolded or misfolded proteins bind to free GRP78, leading to its dissociation from IRE1α, PERK, and ATF6 and facilitating its activation [50]. Our results showed that the expression of GRP78 was up-regulated in DM mice after 12 weeks from the beginning of the experiment, suggesting that ER stress was activated in the STZ group of mice, whereas the expression of GRP78 was reduced after either QKF or MBT treatment. Excessive and sustained ER stress activates the PERK/ATF4 pathway, leading to dissociation of the ATF4/CHOP complex, which in turn activates CHOP. CHOP plays a crucial role in ER stress induced apoptosis [20, 51]. CHOP induces apoptosis by down-regulating Bcl-2 expression and increasing Bax translocation from cytoplasm to mitochondria [52]. At this point in the study, we found that the presence of ER stress in the SN of STZ-induced DPN mice increased the expression of p-PERK and induced the expression of ATF4 and CHOP. In contrast, QKF inhibited the expression of the PERK/ATF4/CHOP pathway in the SN of DPN mice, thereby reducing apoptosis and ameliorating the progression of DPN.

Based on these results, we found that the presence of ER stress in DPN mice, activated by the PERK/ATF4/CHOP pathway, induced an increase in apoptotic protein levels. The therapeutic and preventive effects of QKF in DPN mice may act through this pathway. However further work is needed to fully identify potential targets of QKF in DPN progression. For example, we can use molecular biology techniques such as RNA sequencing and proteomics in the next step to identify genes and proteins whose expression levels change in cells or mice after QKF treatment and then screen for potential targets. Subsequently, the interaction and binding modes between QKF and its targets, as well as the regulatory mechanisms of QKF on the targets, were identified. Finally, methods such as gene editing technology and mouse models can be utilized to validate the biological functions of QKF targets and their regulatory roles in DPN, providing important theoretical and experimental bases for further clinical applications.

## CONCLUSION

**Fig. (8):**
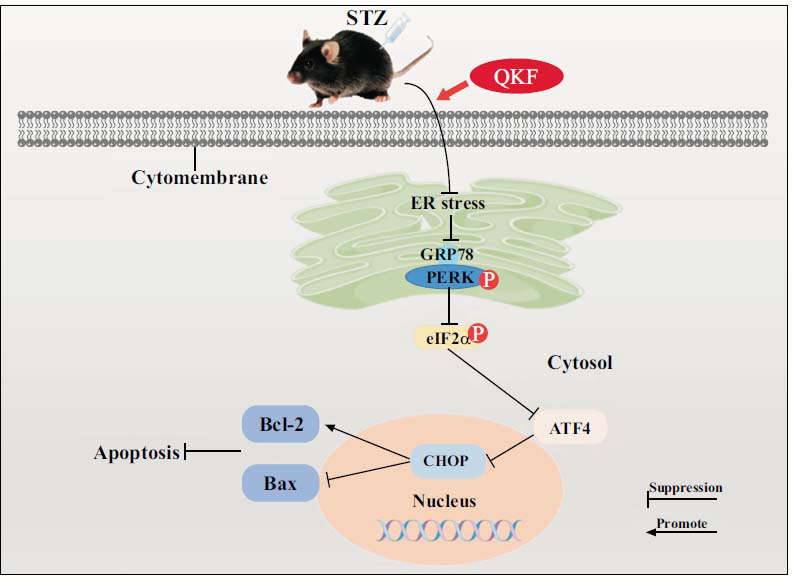
Diagram of the mechanism of QKF in the treatment of DPN.

In the present study, we delineated a total of 69 constituent compounds of QKF utilizing HPLC/QTOF-MS. We conducted a comprehensive exploration of QKF's therapeutic impact on DPN in mice, assessing parameters including body weight, glycemic levels, thermosensitivity, nociception, neural conductance, blood perfusion volume in the footpad tissue and SN region, as well as histopathological alterations in the SN architecture. Meanwhile, the mechanistic aspect of the study confirmed that QKF improved the extent of DPN injury by inhibiting apoptogenesis by inhibiting the PERK/ATF4/CHOP pathway (Fig. **8**).

## Figures and Tables

**Table 1:** Primer sequences for GRP78, ATF4, CHOP, Bcl-2, Bax, and β-actin.

**Genomic**	**Forward Primer**	**Reverse Primer**
GRP78	AGGAGGAGGACAAGAAGGAGGATG	TGAACACACCGACGCAGGAATAG
ATF4	ATGACCCACCTGGAGTTAGTTTGAC	CCTGCTCAGCCCGCTTCTTC
CHOP	CTCGCTCTCCAGATTCCAGTCAG	CGCTCGTTCTCCTGCTCCTTC
Bcl-2	CTACGAGTGGGATGCTGGAGATG	GGTTGCTCTCAGGCTGGAAGG
Bax	GGAGACACCTGAGCTGACCTTG	GCTCCATATTGCTGTCCAGTTCATC
β-actin	GATGGTGGGAATGGGTCAGAAGG	TTGTAGAAGGTGTGGTGCCAGATC

**Table 2:** Body weight levels at 6, 8, 10, and 12 weeks in each group of mice.

**Group**	**6 W**	**8 W**	**10 W**	**12 W**
Ctrl	25.36 ± 0.79	26.96 ± 0.73	28.43 ± 1.11	29.11 ± 0.76
STZ	22.64 ± 1.09^###^	22.49 ± 1.04^###^	21.84 ± 1^###^	20.46 ± 0.94^###^
STZ+QKF (2 g/kg)	23.11 ± 0.72	23.36 ± 0.98	23.26 ± 0.73**	22.76 ± 0.83***
STZ+QKF (4 g/kg)	23.83 ± 0.52*	24.84 ± 0.84***	24.69 ± 0.88***	24 ± 0.58***
STZ+QKF (8 g/kg)	23.49 ± 0.92	24.11 ± 0.89**	24.99 ± 1.07***	24.45 ± 1.33***
STZ+MBT (189 mg/kg)	23.96 ± 1.05*	24.79 ± 0.91***	24.26 ± 0.6***	23.5 ± 1.02***

**Note:** Data were presented as mean ± SEM. #, *versus*. Ctrl group, #*p* < 0.05, ##*p* < 0.01, ###*p* < 0.001; *, *versus*. STZ group, *p < 0.05, **p < 0.01, ***p < 0.001.

## Data Availability

The original contributions presented in the study are included in the article/Supplementary material; further inquiries can be directed to the corresponding author.

## LIST OF ABBREVIATIONS

DM: Diabetes Mellitus
DPN: Diabetic Peripheral Neuropathy
ER: Endoplasmic Reticulum
HPLC: High-performance Liquid Chromatography
LFB: Luxol Fast Blue
LSCI: Laser Speckle Contrast Imaging
MNCV: Motor Nerve Conduction Velocity
MS: Myelin Sheaths
SN: Sciatic Nerve
SNCV: Sensory Nerve Conduction Velocity
STZ: Streptozotocin
TCM: Traditional Chinese Medicine
TEM: Transmission Electron Microscopy
