# Supplementary material for: Qizhi Kebitong Formula Ameliorates Sciatic Nerve Injury in Streptozocin-induced Diabetic Mice through PERK/ATF4/CHOP Endoplasmic Reticulum Stress Signaling Pathway
Source: Curr Pharm Des. 2025 Mar 21;31(29):2370–84. doi: 10.2174/0113816128362557250314054528 (PMC12606609; doi:10.2174/0113816128362557250314054528)
Supplement: Supplementary file 1 [file CPD-31-29-2370_SD1.pdf]

Supplementary Material

Qizhi Kebitong Formula Ameliorates Sciatic Nerve Injury in Streptozocin-induced Diabetic Mice through PERK/ATF4/CHOP Endoplasmic Reticulum Stress Signaling Pathway

Honghai Yu[1,#], Cunqing Yang[2,#], Guoqiang Wang[3], Jiao Lv[1], Xiangyan Li[4,*], Wenxiu Qi[4,*] and Xiuge Wang[3,*]

[1]*College of Traditional Chinese Medicine, Changchun University of Chinese Medicine, Changchun, Jilin, 130117, China;* [2]*Department of Endocrinology, Guang'anmen Hospital, China Academy of Chinese Medical Sciences, Beijing, 100053, China;* [3]*Department of Endocrinology, Affiliated Hospital of Changchun University of Chinese Medicine, Changchun, Jilin, 130012, China;* [4]*Northeast Asia Research Institute of Traditional Chinese Medicine, Key Laboratory of Active Substances and Biological Mechanisms of Ginseng Efficacy, Ministry of Education, Jilin Provincial Key Laboratory of Bio-Macromolecules of Chinese Medicine, Changchun University of Chinese Medicine, Changchun, Jilin, 130117, China*

1873-4286/25 **© 2025 Bentham Science Publishers**

Table S1. QKF chemical composition identification list.

NO	Retention Time (min)	Measured Mass-to-charge Ratio	Precursor Ion/ Summed Ion	Molecular Formula	Theoretical Molecular Weight	Mass Error (ppm)	Secondary Mass Spectrometry Data	Compound Name	Compound Source
1	4.35	315.0723	$[M–H]^-$	$C_{13}H_{16}O_9$	315.0716	2.22	153.0173,135.0456,123.0446,109.0286	Protocatechuic acid 3-O-glucoside	Wei-ling-xian
2	4.59	359.0977	$[M–H]^-$	$C_{15}H_{20}O_{10}$	359.0978	-0.28	197.0451,179.0331,135.0452,123.0449	3-methoxy-4-Hydroxyphenyl glycol glucuronide	Wei-ling-xian
3	4.74	305.0663	$[M–H]^-$	$C_{15}H_{14}O_7$	305.0662	0.33	289.0719,241.0748,179.0348,137.0243	Gallocatechin	Ji-xue-teng
4	5.17	431.1188	$[M–H]^-$	$C_{18}H_{24}O_{12}$	431.119	-0.46	423.0730,152.0079,137.0246,93.0352	Asperulosidic acid	Ji-xue-teng
5	5.20	443.1763	$[M–H]^-$	$C_{17}H_{32}O_{13}$	443.1765	-0.45	205.0879,177.0188,137.0245,93.0352	Lentztrehalose	Niu-xi
6	6.79	475.1455	$[M–H]^-$	$C_{20}H_{28}O_{13}$	475.1452	0.63	327.9642,237.0790,137.0245,123.0453	Primeverin	Ji-xue-teng
7	6.92	447.1137	$[M–H]^-$	$C_{18}H_{24}O_{13}$	447.1139	-0.45	373.1176,179.0322,152.0114,108.0218	4-hydroxy -5-□3',4',5'-trihydroxylphenyl□-valeric acid -O-methyl -O-glucuronide	Wei-ling-xian
8	7.17	375.1294	$[M–H]^-$	$C_{17}H_{20}N_4O_6$	375.1305	-2.93	365.1076,259.9908,135.0446	Riboflavin	Ji-xue-teng
9	8.17	577.1335	$[M–H]^-$	$C_{30}H_{26}O_{12}$	577.1346	-1.91	425.0871,407.0768,289.0716	Procyanidin B1	Ji-xue-teng
10	8.85	291.0855	$[M+H]^+$	$C_{15}H_{14}O_6$	291.0868	-4.47	176.9621,139.0382,123.0437,77.0389	D-(+)-Catechin	Ji-xue-teng
11	8.98	417.1033	$[M–H]^-$	$C_{17}H_{22}O_{12}$	417.1033	0.00	327.1231,241.0703,161.0244,135.0455	Hexamethyl Cyclopentane-1,1,2,2,4,4-Hexacarboxylate	Ji-xue-teng
12	9.17	567.1713	$[M–H]^-$	$C_{26}H_{32}O_{14}$	567.1714	-0.18	405.1184,243.0668,225.0559,175.0765	Mulberroside A	Sang-zhi
13	9.35	329.0514	$[M–H]^-$	$C_{13}H_{14}O_{10}$	329.0509	1.52	197.0458,153.0556,123.0091,121.0300	3,4-Dihydroxybenzoic Acid 3-O-β-D-Glucuronide	Wei-ling-xian
14	10.23	523.1666	$[M–H]^-$	$C_{21}H_{32}O_{15}$	523.1663	0.57	461.1339,305.0671,237.0776,153.0191	Rehmannioside A	Sang-zhi
15	10.51	579.1494	$[M+H]^+$	$C_{30}H_{26}O_{12}$	579.1502	-1.38	449.1170,289.0691,163.0380,127.0384	Procyanidin B2	Ji-xue-teng
16	10.80	865.1965	$[M–H]^-$	$C_{45}H_{38}O_{18}$	865.198	-1.73	695.1333,577.1348,287.0558	procyandin B-type trimer	Ji-xue-teng
17	10.99	325.0561	$[M–H]^-$	$C_{14}H_{14}O_9$	325.056	0.31	193.0503,178.0259,135.0442,134.0369	5-Galloylshikimic acid	Wei-ling-xian
18[a]	11.09	611.1606	$[M–H+FA]^-$	$C_{26}H_{30}O_{14}$	611.1613	-1.15	403.1038,241.0513,177.0181,135.0444	Mulberroside F	Sang-zhi
19	11.20	289.0714	$[M–H]^-$	$C_{15}H_{14}O_6$	289.0712	0.69	245.0813,227.0700,203.0706,187.0387,161.0239	Epicatechin	Ji-xue-teng
20	11.41	485.1639	$[M+H]^+$	$C_{22}H_{28}O_{12}$	485.1659	-4.12	377.1440,219.1004,177.0539,128.0614	3,4-Dihydrocatalposide	Wei-ling-xian
21	11.49	342.1687	$[M+H]^+$	$C_{17}H_{25}O_7$	342.1678	2.63	189.0691,165.0691,152.0612,91.0545	2-Methyl-1-(2-methyl-1,3-dioxolan-2-yl)-5-(tetrahydro-2H-pyran-2-yloxy)-3-hexyn-2-yl carbonate	Wei-ling-xian
22	11.85	561.1395	$[M–H]^-$	$C_{30}H_{26}O_{11}$	561.1397	-0.36	289.0720,245.0832,203.0717,165.0537	(Epi)afzelechin–(epi)catechin	Ji-xue-teng
23	12.04	509.2238	$[M–H]^-$	$C_{22}H_{38}O_{13}$	509.2234	0.79	285.0396,193.0509,134.0376,116.9289	3-(2-methylbutanoyl)-4-(isovaleryloxy)-sucrose	Wei-ling-xian
24[a]	13.27	491.1193	$[M–H+FA]^-$	$C_{22}H_{22}O_{10}$	491.119	0.61	283.0612,255.0295,211.0398,135.0093	Calycosin 7-O-glucoside	Huang-qi
25[a]	13.28	447.1288	$[M+H]^+$	$C_{22}H_{22}O_{10}$	447.1291	-0.67	285.0755,213.0543,137.0232,89.0390	3'-hydroxy-5'-methoxyisoflavone-7-O-	Huang-qi

NO	Retention Time (min)	Measured Mass-to-charge Ratio	Precursor Ion/ Summed Ion	Molecular Formula	Theoretical Molecular Weight	Mass Error (ppm)	Secondary Mass Spectrometry Data	Compound Name	Compound Source
								β-D-glucopyranoside	
26	13.55	177.0545	$[M+H]^+$	$C_{10}H_8O_3$	177.0551	-3.39	151.0379,137.0590,91.0543,77.0393	4-Methylumbelliferone	Wei-ling-xian
27	13.75	525.3062	$[M–H]^–$	$C_{28}H_{46}O_9$	525.3064	-0.38	363.1833,327.0915,283.0610,116.9288	Hythiemoside A	Niu-xi
28	13.76	481.316	$[M+H]^+$	$C_{27}H_{44}O_7$	481.3165	-1.04	194.1067,161.0952	Inokosterone	Niu-xi
29	15.69	187.0974	$[M–H]^–$	$C_9H_{16}O_4$	187.0971	1.60	161.0235,141.0917	Azelaic acid	Ji-xue-teng
30	15.87	533.1293	$[M+H]^+$	$C_{25}H_{24}O_{13}$	533.1295	-0.38	285.0752,270.0514,253.0485,137.0230	Malonylglycitin	Huang-qi
31	16.67	307.0821	$[M–H]^–$	$C_{15}H_{16}O_7$	307.0818	0.98	289.0681,231.9787	Catechin Hydrate	Wei-ling-xian
32	16.67	309.0962	$[M+H]^+$	$C_{15}H_{16}O_7$	309.0974	-3.88	177.0537,134.0359,117.0332,89.0390	Methyl 3-(2-furyl)-3-[3-hydroxy-6-(methoxymethyl)-4-oxo-4H-pyran-2-yl]propanoate	Wei-ling-xian
33	17.32	445.2889	$[M+H]^+$	$C_{34}H_{36}$	445.2895	-1.35	399.1398,259.0941,191.0694,107.0483	3-Hexene, 3,4-bis(1,1'-biphenyl-4-yl)-2,2,5,5-tetramethyl-, (E)	Wei-ling-xian
34	17.50	447.0927	$[M–H]^–$	$C_{21}H_{20}O_{11}$	447.0928	-0.22	283.0602,151.0391	kaempferol-3-O-galactoside	Wei-ling-xian
35	17.99	467.3005	$[M+H]^+$	$C_{26}H_{42}O_7$	467.3009	-0.86	371.1460,341.1355,189.0688,163.0380	Cryptosphaerolide	Ji-xue-teng
36[a]	18.37	475.1242	$[M–H+FA]^–$	$C_{22}H_{22}O_9$	475.1241	0.21	267.0665,223.0386,195.0442,161.0242	Ononin	Huang-qi
37	18.60	489.3132	$[M+H]^+$	$C_{36}H_{40}O$	489.3157	-5.11	255.0636,133.0856,89.0600	1,1'-Biphenyl, 4'-(2,2-diphenylethenyl)-4-[(2-ethylhexyl)oxy]-2,5-dimethyl	Ji-xue-teng
38	19.23	545.2956	$[M–H]^–$	$C_{26}H_{44}O_9$	545.2962	-1.10	299.0555,235.0599,193.1035,171.9451	Ent-2β,15,16,19-tetrahydroxy-pimar-8(14)-en-10-O-β-glucopyranoside	Xi-xian-cao
39	19.56	557.2581	$[M–H+FA]^–$	$C_{26}H_{40}O_{10}$	557.2598	-3.05	349.2005,303.1947,285.0393,243.1718,229.1601	β-D-Glucopyranosyl-ent-2-oxo-15,16-dihydroxy-pimar-8(14)-en-19-oic-late	Xi-xian-cao
40	19.69	431.0975	$[M–H]^–$	$C_{21}H_{20}O_{10}$	431.0978	-0.70	223.0577,216.8526,193.0500,165.0554	Genistin	Wei-ling-xian
41[a]	20.12	301.1067	$[M+H]^+$	$C_{17}H_{16}O_5$	301.1076	-2.99	269.0783,197.0581,167.0695,147.0433	Methylnissolin	Huang-qi
42[a]	20.12	507.1509	$[M–H+FA]^–$	$C_{23}H_{26}O_{10}$	507.1503	1.18	355.0233,299.0922,269.0452,241.0507,116.9291	Methylnissolin-3-O-glucoside	Huang-qi
43	20.78	543.2811	$[M–H+FA]^–$	$C_{26}H_{42}O_9$	543.2806	0.92	533.2521,471.3480,301.1943,247.0826,161.0455	Ent-2-Oxo-3β,15,16-trihydroxy-pimar-8(14)-en-3-O-β-glucopyranoside	Xi-xian-cao
44	20.78	337.2367	$[M+H]^+$	$C_{20}H_{32}O_4$	337.2379	-3.56	185.1313,152.0609,128.0617,105.0697	Ent-2α,15,16,19-tetrahydroxypimar-6,8(14)-diene	Xi-xian-cao
45[a]	21.08	509.1659	$[M–H+FA]^–$	$C_{23}H_{28}O_{10}$	509.1659	0.00	301.1079,271.0610,135.0448,121.0296	Isomucronulatol 7-O-glucoside	Huang-qi
46	21.60	321.2426	$[M+H]^+$	$C_{20}H_{32}O_3$	321.2429	-0.93	269.0802,254.0557,141.0695,128.0681	Ent-17-hydroxy-16βH-kauran-19-oic acid	Xi-xian-cao
47	21.60	383.2432	$[M–H+FA]^–$	$C_{20}H_{34}O_4$	383.2434	-0.52	267.0664,252.0422,195.0449,116.9288	Ent-2α,15,16,19-tetrahydroxypimar-8(14)-ene	Xi-xian-cao
48	22.99	1183.9879	$[M–H]^–$	$C_{70}H_{136}O_{13}$	1183.9903	-2.03	925.4035,749.4475,603.3884,469.1554	Hypsiziprenol-AA14	Xi-xian-cao
49	23.61	349.2014	$[M–H]^–$	$C_{20}H_{30}O_5$	349.2015	-0.29	303.1985,247.0819,205.0718,143.0344	(5S,6Z,8E,10E,12R,14Z)-5,12-dihydroxy-20-oxoicosa-6,8,10,14-	Xi-xian-cao

NO	Retention Time (min)	Measured Mass-to-charge Ratio	Precursor Ion/ Summed Ion	Molecular Formula	Theoretical Molecular Weight	Mass Error (ppm)	Secondary Mass Spectrometry Data	Compound Name	Compound Source
								tetraenoic acid	
50	24.48	327.2174	$[M–H]^–$	$C_{18}H_{32}O_5$	327.2172	0.61	281.0433,241.0019	(10E,15Z)-9,12,13-Trihydroxy-10,15-octadecadienoic acid	Sang-zhi
51	24.73	948.4145	$[M–H+FA]^–$	$C_{50}H_{63}O_{15}$	948.4144	0.11	865.4944,733.4532,469.1562,455.3524	2-O-[β-D-Apiofuranosyl-(1-3)-2-O-(3-methlpentanoyl)-β-D-glucopyranosyl]-4-epi-atractyligenin	Wei-ling-xian
52	24.99	875.3793	$[M–H]^–$	$C_{55}H_{56}O_{10}$	875.3795	-0.23	865.4991,733.4508,587.3944,455.3528	1,5-Anhydro-3,6-di-O-benzyl-2-deoxy-2-formyl-4-O-(2,3,4,6-tetra-O-benzyl-β-D-galactopyranosyl)-D-arabino-hex-1-enitol	Wei-ling-xian
53	25.20	1499.7299	$[M+H]^+$	$C_{70}H_{114}O_{34}$	1499.727	1.93	1083.4796,921.4225,809.2649,603.2098	6-Deoxy-α-L-mannopyranosyl-(1->4)-β-D-glucopyranosyl-(1->6)-1-O-[(3β)-3-{[β-D-glucopyranosyl-(1->4)-β-D-ribopyranosyl-(1->3)-6-deoxy-α-L-mannopyranosyl-(1->2)-α-L-arabinopyrano syl]oxy}-28-oxoolean-12-en-28-yl]-β-D-glucopyranose	Wei-ling-xian
54	26.21	329.2333	$[M–H]^–$	$C_{18}H_{34}O_5$	329.2328	1.52	247.0810,221.0663,161.0446,125.0240	Pinusolidic acid	Huang-qi
55	26.29	1381.6617	$[M–H]^–$	$C_{65}H_{106}O_{31}$	1381.664	-1.66	1043.5167,865.4948,587.3944,455.3520	Hederacholichiside F	Wei-ling-xian
56	26.50	991.5109	$[M–H+FA]^–$	$C_{47}H_{78}O_{19}$	991.5114	-0.50	945.5069,783.4525,651.4168,489.3566	Astragaloside VII	Huang-qi
57	26.97	1117.5067	$[M–H]^–$	$C_{53}H_{82}O_{25}$	1117.5067	0.00	793.4385,731.4376,613.3746,569.3850	Achyranthoside D	Niu-xi
58	26.97	439.3571	$[M+H]^+$	$C_{30}H_{46}O_2$	439.3576	-1.14	393.3496,275.9913,203.1783,191.1784	Ganoderol A	Niu-xi
59[a]	27.81	829.4572	$[M–H+FA]^–$	$C_{41}H_{68}O_{14}$	829.4586	-1.69	783.4505,621.4029,489.3568,383.2933	Astragaloside III	Huang-qi
60	28.08	953.4476	$[M–H]^–$	$C_{47}H_{70}O_{20}$	953.4482	-0.63	793.4374,631.3839,569.3843,455.3525	Bidentatoside I	Niu-xi
61	28.09	439.357	$[M+H]^+$	$C_{30}H_{46}O_2$	439.3576	-1.37	393.3492,289.2137,147.1161	Ganoderic Acid A	Niu-xi
62	28.74	793.4374	$[M–H+FA]^–$	$C_{42}H_{66}O_{14}$	793.4375	-0.13	631.3848,613.3729,455.3531,157.0144	Zingibroside R1	Niu-xi
63	29.20	953.4385	$[M–H]^–$	$C_{47}H_{70}O_{20}$	953.4382	0.31	793.4373,631.3848,613.3742,455.3525	Achyranthoside B	Niu-xi
64	29.63	925.4428	$[M–H]^–$	$C_{60}H_{70}O_{19}$	925.4433	-0.54	673.3896,631.3836,569.3841,455.3525	Achyranthoside E	Niu-xi
65	29.92	516.1962	$[M–H]^–$	$C_{34}H_{29}O_5$	516.1937	4.84	455.3504,241.0022,152.9860,96.9606	2,3,4,5-Tetrakis(phenylmethoxy)phenolate	Niu-xi
66[a]	30.29	871.469	$[M–H+FA]^–$	$C_{43}H_{70}O_{15}$	871.4692	-0.23	837.3696,781.4347,216.8521,171.9473	Cycloastragenol II	Huang-qi
67[a]	30.32	871.4679	$[M–H+FA]^–$	$C_{43}H_{70}O_{15}$	871.4692	-1.49	723.2068,603.3901,489.3555,383.0971	Astragaloside II	Huang-qi
68[a]	32.73	913.4794	$[M–H+FA]^–$	$C_{45}H_{72}O_{16}$	913.4797	-0.33	867.4679,825.4719,807.3806,765.4511	Astragaloside I	Huang-qi
69[a]	32.92	913.4791	$[M–H+FA]^–$	$C_{45}H_{72}O_{16}$	913.4797	-0.66	867.4739,825.4712,807.4513,765.4407	Isoastragaloside I	Huang-qi

Remarks: a is the identification of the standard.
